# Crystal structure of bis­{*N*-[(di­ethyl­amino)­dimethyl­sil­yl]anilido-κ^2^ *N*,*N*′}zinc

**DOI:** 10.1107/S2056989015022768

**Published:** 2015-12-06

**Authors:** Juan Chen

**Affiliations:** a Department of Chemistry, Taiyuan Teachers College, Taiyuan 030031, People’s Republic of China

**Keywords:** crystal structure, zinc amide, N-donor, N—Si—N chelating ligand

## Abstract

The title zinc amide, [Zn(C_12_H_21_N_2_Si)_2_], was prepared by the metathetical reaction of [LiN(SiMe_2_NEt_2_)(C_6_H_5_)]_2_ with zinc dichloride. It is mononuclear and the mol­ecule is generated by twofold rotation symmetry. The central Zn^II^ atom is *N*,*N*′-chelated by each of the two *N*-silylated anilide ligands in a highly distorted tetra­hedral environment. Two N—Si—N ligands are arranged in a *cis* fashion around the Zn^II^ atom. The Zn—N_amine_ bonds [2.2315 (12) Å] are much longer than the Zn—N_anilide_ bonds [1.9367 (11) Å].

## Related literature   

For related compounds which show linear and tetra­hedral coordination, see: Schumann *et al.* (2000 ▸). For applications of zinc amides, see: Armstrong *et al.* (2002 ▸) and for their utility in MOVCD, see Maile & Fischer (2005 ▸). For a related zinc amide with a dimethylanilide ligand instead of an anilide ligand, see: Chen *et al.* (2007 ▸).
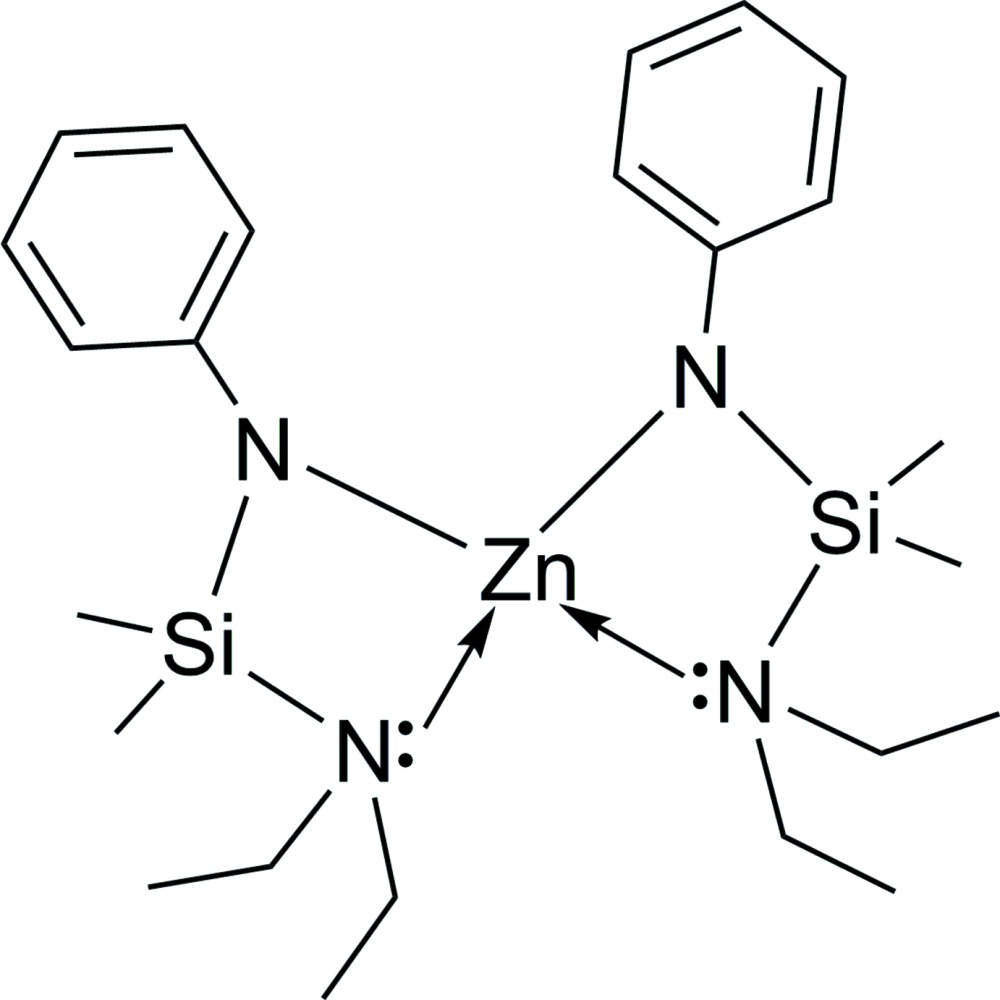


## Experimental   

### Crystal data  

[Zn(C_12_H_21_N_2_Si)_2_]
*M* *_r_* = 508.19
Orthorhombic, 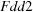

*a* = 29.7954 (12) Å
*b* = 21.3566 (8) Å
*c* = 8.5844 (3) Å
*V* = 5462.5 (4) Å^3^
*Z* = 8
Mo *K*α radiation
μ = 1.01 mm^−1^
*T* = 200 K
0.20 × 0.15 × 0.15 mm

### Data collection  

Bruker SMART area-detector diffractometer
Absorption correction: multi-scan (*SADABS*; Sheldrick, 1996 ▸) *T*_min_ = 0.824, *T*_max_ = 0.864
12927 measured reflections
3296 independent reflections
3153 reflections with *I* > 2σ(*I*)
*R*_int_ = 0.023

### Refinement  

*R*[*F* ^2^ > 2σ(*F* ^2^)] = 0.019
*wR*(*F* ^2^) = 0.051
*S* = 1.12
3296 reflections
147 parameters
1 restraint
H-atom parameters constrained
Δρ_max_ = 0.23 e Å^−3^
Δρ_min_ = −0.23 e Å^−3^
Absolute structure: Flack (1983 ▸), 1484 Friedel pairs
Absolute structure parameter: 0.028 (7)

### 

Data collection: *SMART* (Bruker, 2000 ▸); cell refinement: *SAINT* (Bruker, 2000 ▸); data reduction: *SAINT*; program(s) used to solve structure: *SHELXS97* (Sheldrick, 2008 ▸); program(s) used to refine structure: *SHELXL97* (Sheldrick, 2008 ▸); molecular graphics: *SHELXTL/PC* (Sheldrick, 2008 ▸); software used to prepare material for publication: *SHELXL97*.

## Supplementary Material

Crystal structure: contains datablock(s) I, New_Global_Publ_Block. DOI: 10.1107/S2056989015022768/hp2073sup1.cif

Structure factors: contains datablock(s) I. DOI: 10.1107/S2056989015022768/hp2073Isup2.hkl

**Fig. 1.**
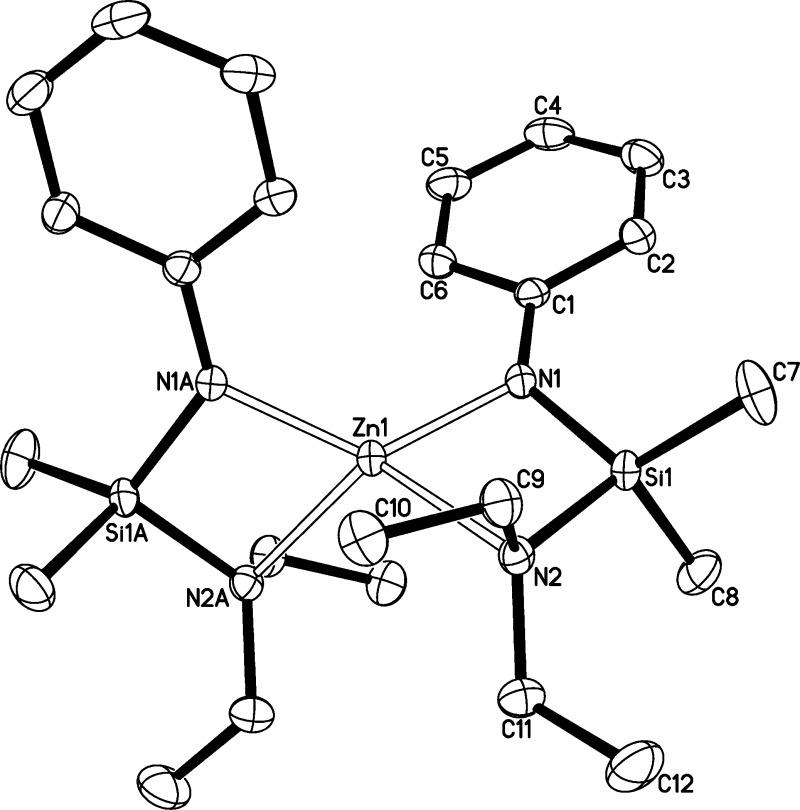
The molecular structure of the title compound, showing the atom-numbering scheme. Displacement ellipsoids are drawn at the 30% probability level. Hydrogen atoms have been omitted for clarity.

. DOI: 10.1107/S2056989015022768/hp2073fig1.tif
The mol­ecular structure of the title compound, showing the atom-numbering scheme. Displacement ellipsoids are drawn at the 30% probability level. Hydrogen atoms have been omitted for clarity.

CCDC reference: 1439385

Additional supporting information:  crystallographic information; 3D view; checkCIF report

## supplementary crystallographic information

### S1. Comment

Zinc amides were good transamination reagents and useful precusors for preparing the zinc thin film through the MOVCD method (Amstrong *et al.*, 2002; Maile *et al.*, 2005).

The title compound was prepared by metathetical reaction of [LiN(SiMe_2_NEt_2_)(C_6_H_5_)]_2_ with zinc dichloride. It is monomeric and similar to the reported bis[(*N*-trimethylsilyl)2,6-dimethylanilido]zinc (Schumann *et al.*, 2000). The ligand fixes Zn center with the N—Si—N chelating unit, giving an N—Zn—N bite angle of 76.98°. The N—Si—N group is presumed to be a "quasi" conjugated system owing to *d*—*π* interaction between Si and N atoms, but is much more flexible in contrast to the rigid N—C—N chelating unit in the amidinate ligand. The Zn—N_anilide_ bonds are in the normal range. The Zn—N_amine_ bonds are about 0.3 Å longer than the Zn—N_anilide_ bonds. Two N—Si—N ligands are arranged in a *cis* fashion around Zn, composing a highly distorted tetrahedral environment. The situation is quite different from an analgous zinc amide with the similar ligand, in which the two ligands are *trans* to each other (Chen *et al.*, 2007). Two types of ligands have slightly different steric effect.

### S2. Experimental

A solution of LiBu^*n*^ (2.2 *M*, 2.27 ml, 5.0 mmol) in hexane was slowly added into a solution of [NH(SiMe_2_NEt_2_)(C_6_H_5_)]_2_ (1.14 g, 5.0 mmol) in Et_2_O (30 ml) at 273 K by syringe. The mixture was stirred at room temperature for five hours and then ZnCl_2_ (0.56 g, 2.5 mmol) was added at 273 K. The resulting solution was stirred at room temperature overnight. The filtrate was concentrated to give the title compound as colorless crystals (yield 0.85 g, 67%).

### S3. Refinement

The methyl H atoms were constrained to an ideal geometry, with C—H distances of 0.98 Å and *U*_iso_(H) = 1.5*U*_eq_(C), but each group was allowed to rotate freely along its C—C bond. The methylene H atoms were constrained with C—H distances of 0.99 Å and *U*_iso_(H) = 1.2*U*_eq_(C). The phenyl H atoms were placed in geometrically idealized positions and constrained to ride on their parent atoms, with C—H distances in the range 0.95 Å and *U*_iso_(H) = 1.2*U*_eq_(C).

### Crystal data

**Table:**

[Zn(C_12_H_21_N_2_Si)_2_]	*F*(000) = 2176
*M**_r_* = 508.19	*D*_x_ = 1.236 Mg m^−^^3^
Orthorhombic, *F**d**d*2	Mo *K*α radiation, λ = 0.71073 Å
Hall symbol: F 2 -2d	Cell parameters from 9968 reflections
*a* = 29.7954 (12) Å	θ = 2.8–28.3°
*b* = 21.3566 (8) Å	µ = 1.01 mm^−^^1^
*c* = 8.5844 (3) Å	*T* = 200 K
*V* = 5462.5 (4) Å^3^	Block, colorless
*Z* = 8	0.20 × 0.15 × 0.15 mm

### Data collection

**Table:**

Bruker SMART area-detector diffractometer	3296 independent reflections
Radiation source: fine-focus sealed tube	3153 reflections with *I* > 2σ(*I*)
Graphite monochromator	*R*_int_ = 0.023
φ and ω scan	θ_max_ = 28.3°, θ_min_ = 3.3°
Absorption correction: multi-scan (*SADABS*; Sheldrick, 1996)	*h* = −39→36
*T*_min_ = 0.824, *T*_max_ = 0.864	*k* = −28→28
12927 measured reflections	*l* = −11→11

### Refinement

**Table:**

Refinement on *F*^2^	Hydrogen site location: inferred from neighbouring sites
Least-squares matrix: full	H-atom parameters constrained
*R*[*F*^2^ > 2σ(*F*^2^)] = 0.019	*w* = 1/[σ^2^(*F*_o_^2^) + (0.0141*P*)^2^ + 1.7098*P*] where *P* = (*F*_o_^2^ + 2*F*_c_^2^)/3
*wR*(*F*^2^) = 0.051	(Δ/σ)_max_ < 0.001
*S* = 1.12	Δρ_max_ = 0.23 e Å^−^^3^
3296 reflections	Δρ_min_ = −0.23 e Å^−^^3^
147 parameters	Extinction correction: *SHELXL97* (Sheldrick, 2008), Fc^*^=kFc[1+0.001xFc^2^λ^3^/sin(2θ)]^-1/4^
1 restraint	Extinction coefficient: 0.00049 (6)
Primary atom site location: structure-invariant direct methods	Absolute structure: Flack (1983), 1484 Friedel pairs
Secondary atom site location: difference Fourier map	Absolute structure parameter: 0.028 (7)

### Special details

**Table:**

Geometry. All e.s.d.'s (except the e.s.d. in the dihedral angle between two l.s. planes) are estimated using the full covariance matrix. The cell e.s.d.'s are taken into account individually in the estimation of e.s.d.'s in distances, angles and torsion angles; correlations between e.s.d.'s in cell parameters are only used when they are defined by crystal symmetry. An approximate (isotropic) treatment of cell e.s.d.'s is used for estimating e.s.d.'s involving l.s. planes.
Refinement. Refinement of *F*^2^ against ALL reflections. The weighted *R*-factor *wR* and goodness of fit *S* are based on *F*^2^, conventional *R*-factors *R* are based on *F*, with *F* set to zero for negative *F*^2^. The threshold expression of *F*^2^ > σ(*F*^2^) is used only for calculating *R*-factors(gt) *etc*. and is not relevant to the choice of reflections for refinement. *R*-factors based on *F*^2^ are statistically about twice as large as those based on *F*, and *R*-factors based on ALL data will be even larger.

### Fractional atomic coordinates and isotropic or equivalent isotropic displacement parameters (Å^2^)

**Table:**

	*x*	*y*	*z*	*U*_iso_*/*U*_eq_	
Zn1	0.2500	0.2500	0.29180 (2)	0.02397 (7)	
Si1	0.247147 (12)	0.121100 (17)	0.32504 (4)	0.02649 (10)	
N2	0.28157 (4)	0.17456 (5)	0.43443 (15)	0.0259 (2)	
N1	0.22836 (4)	0.17412 (5)	0.19372 (15)	0.0272 (2)	
C1	0.20137 (5)	0.16863 (6)	0.06303 (15)	0.0270 (3)	
C2	0.19263 (5)	0.11107 (7)	−0.01073 (18)	0.0342 (3)	
H2	0.2063	0.0740	0.0281	0.041*	
C3	0.16452 (6)	0.10739 (8)	−0.1389 (2)	0.0418 (4)	
H3	0.1592	0.0679	−0.1863	0.050*	
C6	0.18084 (5)	0.22167 (7)	−0.00242 (17)	0.0348 (3)	
H6	0.1864	0.2616	0.0424	0.042*	
C8	0.20082 (6)	0.08844 (8)	0.4469 (2)	0.0443 (4)	
H8A	0.1845	0.1228	0.4971	0.066*	
H8B	0.2134	0.0608	0.5268	0.066*	
H8C	0.1802	0.0646	0.3807	0.066*	
C9	0.32965 (5)	0.17499 (7)	0.38603 (19)	0.0329 (3)	
H9A	0.3313	0.1681	0.2721	0.039*	
H9B	0.3453	0.1397	0.4375	0.039*	
C11	0.27592 (6)	0.17628 (8)	0.60640 (19)	0.0359 (3)	
H11A	0.2434	0.1768	0.6303	0.043*	
H11B	0.2888	0.2159	0.6458	0.043*	
C7	0.28096 (6)	0.05379 (8)	0.2524 (3)	0.0530 (5)	
H7A	0.2609	0.0192	0.2244	0.080*	
H7B	0.3017	0.0400	0.3342	0.080*	
H7C	0.2981	0.0667	0.1605	0.080*	
C4	0.14411 (5)	0.15989 (9)	−0.19904 (19)	0.0432 (4)	
H4	0.1245	0.1568	−0.2860	0.052*	
C12	0.29757 (7)	0.12168 (10)	0.6950 (3)	0.0568 (5)	
H12A	0.2863	0.0819	0.6535	0.085*	
H12B	0.2900	0.1248	0.8059	0.085*	
H12C	0.3302	0.1234	0.6824	0.085*	
C10	0.35400 (5)	0.23565 (7)	0.4258 (2)	0.0401 (4)	
H10A	0.3383	0.2710	0.3776	0.060*	
H10B	0.3848	0.2337	0.3862	0.060*	
H10C	0.3545	0.2412	0.5391	0.060*	
C5	0.15272 (6)	0.21749 (8)	−0.12999 (19)	0.0402 (4)	
H5	0.1392	0.2543	−0.1708	0.048*	

### Atomic displacement parameters (Å^2^)

**Table:**

	*U*^11^	*U*^22^	*U*^33^	*U*^12^	*U*^13^	*U*^23^
Zn1	0.02833 (11)	0.01733 (9)	0.02623 (11)	−0.00051 (8)	0.000	0.000
Si1	0.0303 (2)	0.01746 (17)	0.0317 (3)	0.00078 (13)	−0.00043 (17)	0.00132 (13)
N2	0.0254 (6)	0.0237 (5)	0.0286 (6)	0.0029 (4)	−0.0020 (5)	0.0004 (5)
N1	0.0366 (7)	0.0197 (5)	0.0253 (5)	−0.0027 (5)	−0.0024 (5)	−0.0004 (5)
C1	0.0303 (7)	0.0291 (7)	0.0216 (7)	−0.0042 (5)	0.0044 (5)	−0.0004 (5)
C2	0.0417 (8)	0.0294 (7)	0.0316 (7)	−0.0058 (6)	0.0021 (6)	−0.0041 (6)
C3	0.0474 (10)	0.0449 (9)	0.0332 (8)	−0.0118 (7)	0.0002 (7)	−0.0115 (7)
C6	0.0453 (9)	0.0326 (7)	0.0264 (7)	0.0006 (6)	−0.0035 (6)	−0.0019 (6)
C8	0.0461 (10)	0.0440 (9)	0.0427 (9)	−0.0117 (7)	0.0022 (8)	0.0132 (8)
C9	0.0279 (7)	0.0294 (7)	0.0414 (8)	0.0040 (5)	0.0005 (6)	−0.0020 (6)
C11	0.0372 (8)	0.0418 (9)	0.0286 (7)	0.0022 (6)	−0.0030 (6)	0.0028 (6)
C7	0.0506 (10)	0.0278 (7)	0.0806 (15)	0.0098 (7)	−0.0018 (10)	−0.0167 (9)
C4	0.0396 (9)	0.0651 (11)	0.0250 (7)	−0.0074 (7)	−0.0038 (7)	−0.0055 (8)
C12	0.0654 (12)	0.0608 (12)	0.0442 (10)	0.0046 (9)	−0.0136 (10)	0.0175 (9)
C10	0.0296 (8)	0.0406 (8)	0.0502 (10)	−0.0015 (6)	−0.0034 (7)	−0.0047 (7)
C5	0.0442 (9)	0.0499 (10)	0.0267 (7)	0.0068 (7)	−0.0021 (6)	0.0027 (7)

### Geometric parameters (Å, º)

**Table:**

Zn1—N1	1.9367 (11)	C8—H8B	0.9800
Zn1—N1^i^	1.9367 (11)	C8—H8C	0.9800
Zn1—N2^i^	2.2315 (12)	C9—C10	1.524 (2)
Zn1—N2	2.2315 (12)	C9—H9A	0.9900
Zn1—Si1^i^	2.7689 (4)	C9—H9B	0.9900
Si1—N1	1.6930 (13)	C11—C12	1.535 (2)
Si1—N2	1.7993 (12)	C11—H11A	0.9900
Si1—C7	1.8628 (16)	C11—H11B	0.9900
Si1—C8	1.8669 (17)	C7—H7A	0.9800
N2—C11	1.486 (2)	C7—H7B	0.9800
N2—C9	1.4917 (18)	C7—H7C	0.9800
N1—C1	1.3854 (18)	C4—C5	1.389 (2)
C1—C6	1.405 (2)	C4—H4	0.9500
C1—C2	1.4070 (19)	C12—H12A	0.9800
C2—C3	1.385 (2)	C12—H12B	0.9800
C2—H2	0.9500	C12—H12C	0.9800
C3—C4	1.376 (3)	C10—H10A	0.9800
C3—H3	0.9500	C10—H10B	0.9800
C6—C5	1.382 (2)	C10—H10C	0.9800
C6—H6	0.9500	C5—H5	0.9500
C8—H8A	0.9800		
			
N1—Zn1—N1^i^	128.46 (8)	Si1—C8—H8C	109.5
N1—Zn1—N2^i^	134.62 (5)	H8A—C8—H8C	109.5
N1^i^—Zn1—N2^i^	76.98 (5)	H8B—C8—H8C	109.5
N1—Zn1—N2	76.98 (5)	N2—C9—C10	113.59 (12)
N1^i^—Zn1—N2	134.62 (5)	N2—C9—H9A	108.8
N2^i^—Zn1—N2	113.45 (6)	C10—C9—H9A	108.8
N1—Zn1—Si1^i^	152.49 (4)	N2—C9—H9B	108.8
N1^i^—Zn1—Si1^i^	37.12 (4)	C10—C9—H9B	108.8
N2^i^—Zn1—Si1^i^	40.41 (3)	H9A—C9—H9B	107.7
N2—Zn1—Si1^i^	130.41 (3)	N2—C11—C12	115.22 (15)
N1—Si1—N2	96.41 (6)	N2—C11—H11A	108.5
N1—Si1—C7	118.18 (9)	C12—C11—H11A	108.5
N2—Si1—C7	110.86 (7)	N2—C11—H11B	108.5
N1—Si1—C8	112.23 (8)	C12—C11—H11B	108.5
N2—Si1—C8	111.48 (7)	H11A—C11—H11B	107.5
C7—Si1—C8	107.39 (9)	Si1—C7—H7A	109.5
C11—N2—C9	112.67 (12)	Si1—C7—H7B	109.5
C11—N2—Si1	118.00 (11)	H7A—C7—H7B	109.5
C9—N2—Si1	113.95 (9)	Si1—C7—H7C	109.5
C11—N2—Zn1	118.64 (10)	H7A—C7—H7C	109.5
C9—N2—Zn1	104.34 (8)	H7B—C7—H7C	109.5
Si1—N2—Zn1	86.07 (5)	C3—C4—C5	118.69 (15)
C1—N1—Si1	132.42 (10)	C3—C4—H4	120.7
C1—N1—Zn1	128.03 (9)	C5—C4—H4	120.7
Si1—N1—Zn1	99.21 (6)	C11—C12—H12A	109.5
N1—C1—C6	120.57 (12)	C11—C12—H12B	109.5
N1—C1—C2	123.07 (13)	H12A—C12—H12B	109.5
C6—C1—C2	116.36 (13)	C11—C12—H12C	109.5
C3—C2—C1	121.27 (15)	H12A—C12—H12C	109.5
C3—C2—H2	119.4	H12B—C12—H12C	109.5
C1—C2—H2	119.4	C9—C10—H10A	109.5
C4—C3—C2	121.25 (15)	C9—C10—H10B	109.5
C4—C3—H3	119.4	H10A—C10—H10B	109.5
C2—C3—H3	119.4	C9—C10—H10C	109.5
C5—C6—C1	121.94 (15)	H10A—C10—H10C	109.5
C5—C6—H6	119.0	H10B—C10—H10C	109.5
C1—C6—H6	119.0	C6—C5—C4	120.48 (16)
Si1—C8—H8A	109.5	C6—C5—H5	119.8
Si1—C8—H8B	109.5	C4—C5—H5	119.8
H8A—C8—H8B	109.5		
			
N1—Si1—N2—C11	129.24 (11)	N1^i^—Zn1—N1—C1	−40.18 (11)
C7—Si1—N2—C11	−107.31 (13)	N2^i^—Zn1—N1—C1	72.13 (15)
C8—Si1—N2—C11	12.27 (14)	N2—Zn1—N1—C1	−177.65 (13)
N1—Si1—N2—C9	−95.22 (10)	Si1^i^—Zn1—N1—C1	7.03 (19)
C7—Si1—N2—C9	28.23 (13)	N1^i^—Zn1—N1—Si1	145.83 (7)
C8—Si1—N2—C9	147.82 (10)	N2^i^—Zn1—N1—Si1	−101.86 (7)
N1—Si1—N2—Zn1	8.73 (6)	N2—Zn1—N1—Si1	8.36 (6)
C7—Si1—N2—Zn1	132.18 (8)	Si1^i^—Zn1—N1—Si1	−166.96 (3)
C8—Si1—N2—Zn1	−108.23 (7)	Si1—N1—C1—C6	162.62 (12)
N1—Zn1—N2—C11	−127.69 (11)	Zn1—N1—C1—C6	−9.3 (2)
N1^i^—Zn1—N2—C11	100.35 (13)	Si1—N1—C1—C2	−17.0 (2)
N2^i^—Zn1—N2—C11	5.59 (10)	Zn1—N1—C1—C2	171.08 (11)
Si1^i^—Zn1—N2—C11	49.47 (12)	N1—C1—C2—C3	178.36 (14)
N1—Zn1—N2—C9	105.95 (9)	C6—C1—C2—C3	−1.3 (2)
N1^i^—Zn1—N2—C9	−26.01 (12)	C1—C2—C3—C4	0.0 (2)
N2^i^—Zn1—N2—C9	−120.77 (9)	N1—C1—C6—C5	−178.12 (14)
Si1^i^—Zn1—N2—C9	−76.89 (9)	C2—C1—C6—C5	1.5 (2)
N1—Zn1—N2—Si1	−7.78 (5)	C11—N2—C9—C10	−66.02 (17)
N1^i^—Zn1—N2—Si1	−139.74 (6)	Si1—N2—C9—C10	156.06 (12)
N2^i^—Zn1—N2—Si1	125.50 (5)	Zn1—N2—C9—C10	63.99 (14)
Si1^i^—Zn1—N2—Si1	169.383 (19)	C9—N2—C11—C12	−59.38 (18)
N2—Si1—N1—C1	176.23 (13)	Si1—N2—C11—C12	76.70 (17)
C7—Si1—N1—C1	58.42 (16)	Zn1—N2—C11—C12	178.35 (12)
C8—Si1—N1—C1	−67.40 (16)	C2—C3—C4—C5	1.0 (2)
N2—Si1—N1—Zn1	−10.18 (7)	C1—C6—C5—C4	−0.5 (2)
C7—Si1—N1—Zn1	−127.99 (8)	C3—C4—C5—C6	−0.7 (2)
C8—Si1—N1—Zn1	106.18 (8)		

Symmetry code: (i) −*x*+1/2, −*y*+1/2, *z*.
